# Supplementary material for: Map7D2 and Map7D1 facilitate microtubule stabilization through distinct mechanisms in neuronal cells
Source: Life Sci Alliance. 2022 Apr 25;5(8):e202201390. doi: 10.26508/lsa.202201390 (PMC9039348; doi:10.26508/lsa.202201390)

Kikuchi_Source data figure for Fig. 2

Fig. 2B

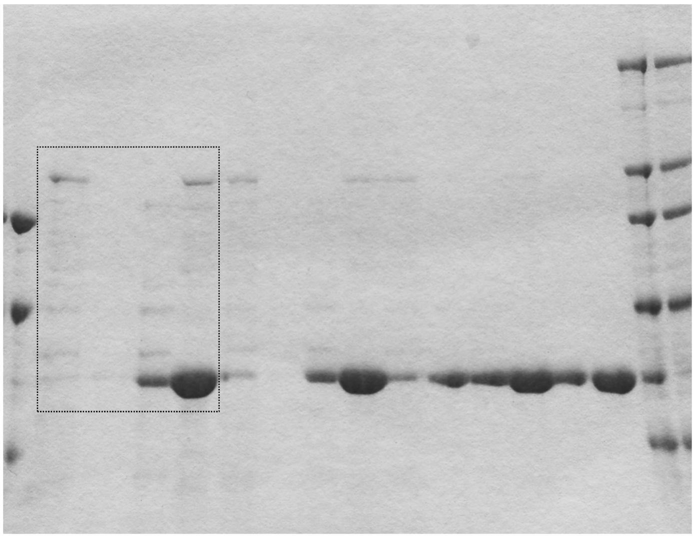

GST-rMap7D2 (µM)	Bound GST-rMap7D2/Tubulin dimer (mol/mol)
0	0
0.184976946	0.050344055
0.277465418	0.07373998
0.414515183	0.098053393
0.545743664	0.108145752
0.643052919	0.110898214

Bound (µM)	bound/free
0.403266233	0.627112049
0.393257281	0.720589734
0.356557791	0.860180291
0.268145382	0.966410099
0.183069291	0.989687069

Fig. 2C

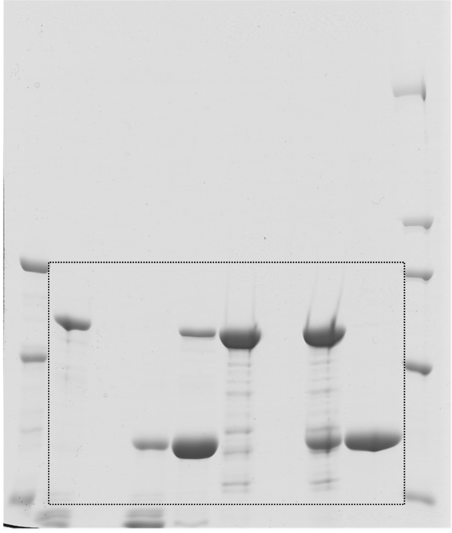

Supplement: Supplementary file 1 [file LSA-2022-01390_SdataF2.1.pdf]
