# Supplementary figures and images for: Map7D2 and Map7D1 facilitate microtubule stabilization through distinct mechanisms in neuronal cells
Source: Life Sci Alliance. 2022 Apr 25;5(8):e202201390. doi: 10.26508/lsa.202201390 (PMC9039348; doi:10.26508/lsa.202201390)

## Kikuchi\_Source data figure for Fig. 3

Fig. 3D

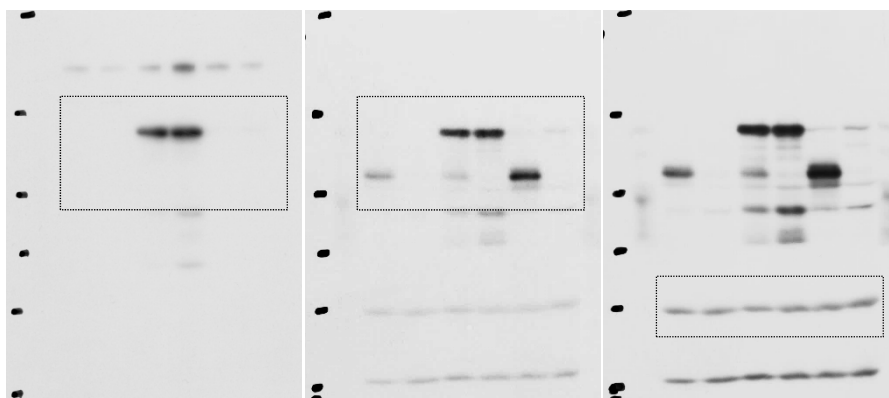

Supplement: Supplementary file 3 [file LSA-2022-01390_SdataF3.pdf]
