# Supplementary material for: Map7D2 and Map7D1 facilitate microtubule stabilization through distinct mechanisms in neuronal cells
Source: Life Sci Alliance. 2022 Apr 25;5(8):e202201390. doi: 10.26508/lsa.202201390 (PMC9039348; doi:10.26508/lsa.202201390)
Supplement: Supplementary file 4 [file LSA-2022-01390_SdataF4.1.pdf]

Kikuchi_Source data figure for Fig. 4

Fig. 4A

	Control		si*Map7d1*		si*Map7d2*	
	0 ng/mL	10 ng/mL	0 ng/mL	10 ng/mL	0 ng/mL	10 ng/mL
1	100	92.85714286	95.12195122	12.5	89.47368421	9.090909091
2	100	90.69767442	93.33333333	7.142857143	91.66666667	9.523809524
3	100	97.2972973	92.85714286	14.28571429	93.10344828	8.333333333
Average	100	93.61737152	93.77080914	11.30952381	91.41459972	8.982683983
SD	0	3.364850109	1.194101352	3.717260713	1.827963376	0.602571901

Fig. 4B

EB1	Control	si*Map7d1*	si*Map7d2*
95%	1.431928318	2.009084569	1.646897385
Quartile-3	1.258686201	1.578753807	1.485005478
Median	1	1.26863471	1.219937748
Quartile-1	0.701939511	0.872003085	1.054691823
5%	0.497362643	0.72945425	0.781439535

Kif5b	Control	si*Map7d1*	si*Map7d2*
95%	0.334284742	0.202402725	0.27534057
Quartile-3	0.132215503	0.120701968	0.138139531
Median	0.070767769	0.06972927	0.068319174
Quartile-1	0.031751467	0.036625105	0.034744387
5%	0.007785746	0.009365678	0.013859822
