# Supplementary material for: Map7D2 and Map7D1 facilitate microtubule stabilization through distinct mechanisms in neuronal cells
Source: Life Sci Alliance. 2022 Apr 25;5(8):e202201390. doi: 10.26508/lsa.202201390 (PMC9039348; doi:10.26508/lsa.202201390)
Supplement: Supplementary file 6 [file LSA-2022-01390_SdataF5.1.pdf]

Kikuchi_Source data figure for Fig. 5

Fig. 5A

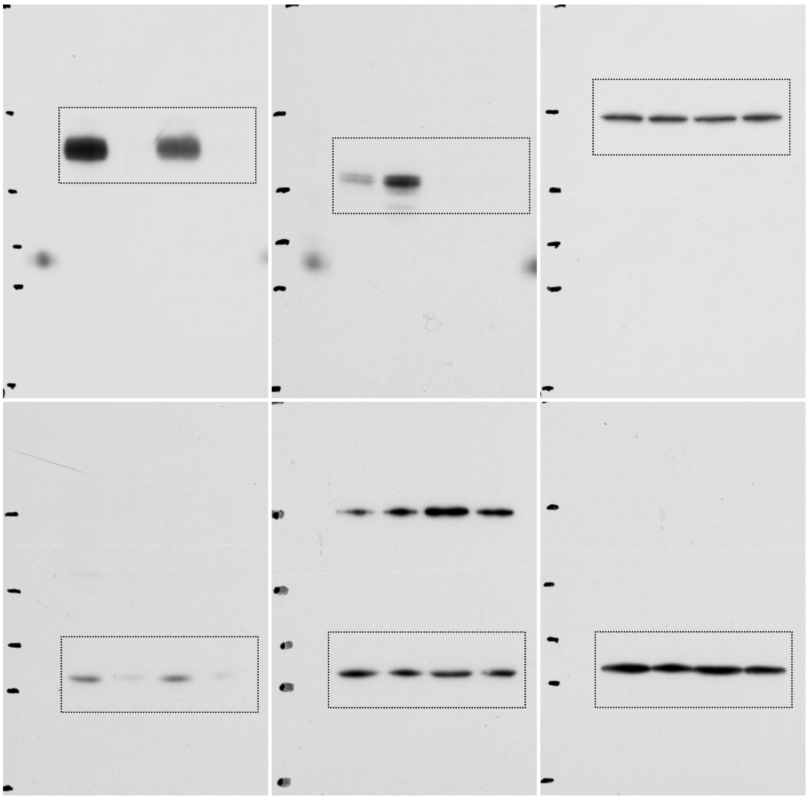

Fig. 5B

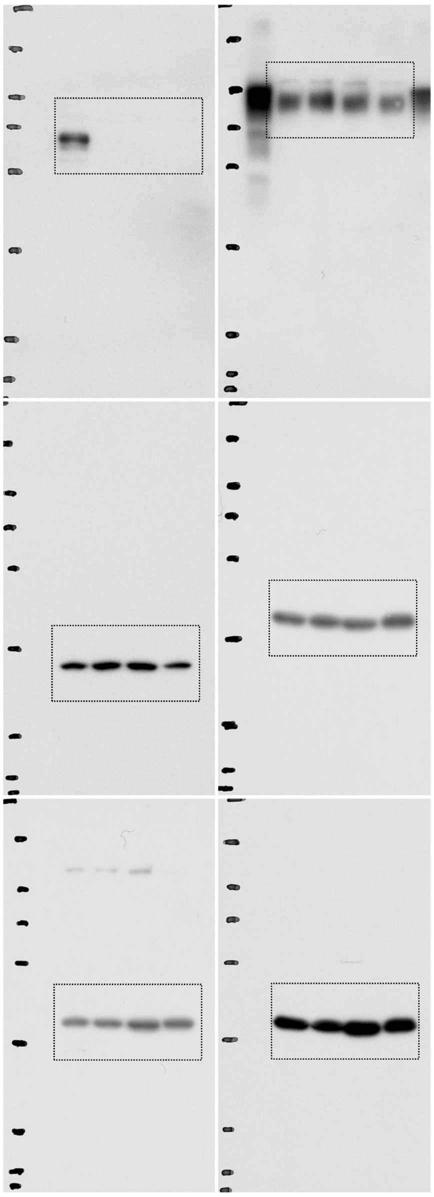

Fig. 5D

Control	Acetylated	Map7D1	α-Tubulin
95%	2.327814452	2.047413	2.288703289
Quartile-3	1.567823439	1.329987891	1.421366731
Median	1	1	1
Quartile-1	0.686221367	0.714267143	0.718608256
5%	0.442114707	0.538681709	0.466957527

si*Map7d2*	Acetylated	Map7D1	α-Tubulin
95%	2.201045261	1.291476632	1.317658251
Quartile-3	1.499565419	0.851067506	0.916445753
Median	1.018438128	0.677778423	0.748488754
Quartile-1	0.652483287	0.561641844	0.549647508
5%	0.35716493	0.389519638	0.330679796

Control	Acetylated	Map7D1	α-Tubulin
95%	2.165795724	2.143109339	2.978828511
Quartile-3	1.428444181	1.329584282	1.467083375
Median	1	1	1
Quartile-1	0.706428147	0.811218679	0.771347918
5%	0.410852138	0.621725513	0.460086702

si*Map7d1*	Acetylated	Map7D1	α-Tubulin
95%	0.634553147	2.571882118	1.721695735
Quartile-3	0.37481443	1.700242027	0.98628894
Median	0.241166865	1.275199317	0.72809759
Quartile-1	0.155247922	1.021853645	0.527674161
5%	0.085013361	0.808001139	0.257989717
