# Supplementary material for: Map7D2 and Map7D1 facilitate microtubule stabilization through distinct mechanisms in neuronal cells
Source: Life Sci Alliance. 2022 Apr 25;5(8):e202201390. doi: 10.26508/lsa.202201390 (PMC9039348; doi:10.26508/lsa.202201390)
Supplement: Supplementary file 8 [file LSA-2022-01390_SdataF6.1.pdf]

Kikuchi_Source data figure for Fig. 6

Fig. 6C

Velocity	Control	si*Map7d1*	si*Map7d2*	si*Map7d1/d2*	*Map7d2*$^{-/-}$
95%	2.28	2.45	2.95	2.64	2.63
Quartile-3	1.42	1.79	2.13	1.93	1.99
Median	1.10	1.38	1.56	1.68	1.53
Quartile-1	0.83	1.09	1.12	1.39	1.19
5%	0.56	0.83	0.87	1.00	0.78

Net distance	Control	si*Map7d1*	si*Map7d2*	si*Map7d1/d2*	*Map7d2*$^{-/-}$
95%	376.06	397.14	500.29	450.12	440.50
Quartile-3	234.05	288.74	324.73	331.97	311.48
Median	177.73	229.45	252.98	265.24	230.56
Quartile-1	133.53	175.60	187.45	215.71	168.82
5%	75.02	138.83	114.56	166.24	120.19

Fig. 6D

	Control	si*Map7d1*	si*Map7d2*	si*Map7d1/d2*	*Map7d2*$^{-/-}$
1	51.68539326	67.23163842	69.28571429	81.43459916	73.26732673
2	56.94444444	61.84210526	65.32258065	86.33879781	77.73722628
3	51.02040816	62.19512195	71.42857143	82.88288288	70.25316456
4	48.17518248	69.87951807	69.02654867	83.45864662	-
Average	51.95635709	65.28709593	68.76585376	83.52873162	73.75257252
SD	3.657308604	3.928521792	2.535377153	2.057822092	3.765553421
