# Supplementary material for: Map7D2 and Map7D1 facilitate microtubule stabilization through distinct mechanisms in neuronal cells
Source: Life Sci Alliance. 2022 Apr 25;5(8):e202201390. doi: 10.26508/lsa.202201390 (PMC9039348; doi:10.26508/lsa.202201390)
Supplement: Supplementary file 10 [file LSA-2022-01390_TableS1.docx]

**Table S1 - Primary antibodies used in this study.**

| Company | Name, catalog number | Used for (dilutions) |
| --- | --- | --- |
| BD Biosciences | Mouse anti-Clathrin heavy chain, 610500 | IB (1:5000) |
|  | Mouse anti-EB1(5/EB1), 610534, | IF (1:500) |
| GeneTex | Rabbit anti-Kif5b, GTX104874 | IF (1:500) |
| Millipore | Rabbit anti-Detyrosinated tubulin, AB3201 | IB (1:3000) |
| MP Biomedicals | Mouse anti-Actin (C4), 0869100-CF | IB (1:10000) |
| Nacalai | Mouse anti-V5, 04434-36 | IB (1:3000) |
|  | Rat anti-GFP, 04404-84 | IB (1:3000) |
| Sigma-Aldrich | Mouse anti-Acetylated tubulin, T7451 | IB (1:5000), IF (1:250) |
|  | Mouse anti-α-tubulin (DM1A), T6199 | IB (1:10000), IF (1:500) |
|  | Mouse anti-γ-tubulin (GTU-88), T6557 | IB (1:5000) |
|  | Mouse anti-Map2, | IF (1:250) |
|  | Mouse anti-Tubb3 | IF (1:250) |
| Made in-house | Rabbit anti-Map7D1 ([Kikuchi et al 2018](#_ENREF_1)) | IB (1:10000), IF (1:500) |
|  | Rabbit anti-Map7D2 | IB (1:10000), IF (1:500) |
|  | Mouse anti-Myc monoclonal (9E10) | IF (1:250) |

IB, Immunoblotting; IF, Immunofluorescence

**References**

Kikuchi K, Nakamura A, Arata M, Shi D, Nakagawa M, Tanaka T, Uemura T, Fujimori T, Kikuchi A, Uezu A, et al. 2018. Map7/7d1 and dvl form a feedback loop that facilitates microtubule remodeling and wnt5a signaling. EMBO Rep. 19(7):e45471.
