# Supplementary material for: Map7D2 and Map7D1 facilitate microtubule stabilization through distinct mechanisms in neuronal cells
Source: Life Sci Alliance. 2022 Apr 25;5(8):e202201390. doi: 10.26508/lsa.202201390 (PMC9039348; doi:10.26508/lsa.202201390)
Supplement: Supplementary file 11 [file LSA-2022-01390_TableS2.docx]

**Table S2 - siRNAs used in this study.**

| Name | Sequences |
| --- | --- |
| si*Luc* (+) | CGUACGCGGAAUACUUCGAAAUGUC |
| si*Luc* (-) | GACAUUUCGAAGUAUUCCGCGUACG |
| si*Map7d1*-1_380 (+) | UUUACAUCUUGCUUGGCAGGAGGGC |
| si*Map7d1*-1_380 (-) | GCCCUCCUGCCAAGCAAGAUGUAAA |
| si*Map7d1*-2_2144 (+) | UUCAUUAUCUCCUCCAGCCGCUUUC |
| si*Map7d1*-2_2144 (-) | GAAAGCGGCUGGAGGAGAUAAUGAA |
| si*Map7d1*-3_2157 (+) | UUUCCGAGUCCUCUUCAUUAUCUCC |
| si*Map7d1*-3_2157 (-) | GGAGAUAAUGAAGAGGACUCGGAAA |
| si*Map7d2*-1_1898 (+) | AACACAUUGAUUUCGAUCUUGUUGG |
| si*Map7d2*-1_1898 (-) | CCAACAAGAUCGAAAUCAAUGUGUU |
| si*Map7d2*-2_2062 (+) | UAGACUGAACGUCUUCAGUUGAGUC |
| si*Map7d2*-2_2062 (-) | GACUCAACUGAAGACGUUCAGUCUA |
| si*Map7d2*-3_2320 (+) | AACAGAAGGUAUUCAGGGUAGUUUC |
| si*Map7d2*-3_2320 (-) | GAAACUACCCUGAAUACCUUCUGUU |

**References**

Kikuchi K, Nakamura A, Arata M, Shi D, Nakagawa M, Tanaka T, Uemura T, Fujimori T, Kikuchi A, Uezu A, et al. 2018. Map7/7d1 and dvl form a feedback loop that facilitates microtubule remodeling and wnt5a signaling. EMBO Rep. 19(7):e45471.
