# Supplementary material for: Map7D2 and Map7D1 facilitate microtubule stabilization through distinct mechanisms in neuronal cells
Source: Life Sci Alliance. 2022 Apr 25;5(8):e202201390. doi: 10.26508/lsa.202201390 (PMC9039348; doi:10.26508/lsa.202201390)
Supplement: Supplementary file 12 [file LSA-2022-01390_TableS3.docx]

**Table S3 - Primers for RT-qPCR in N1E115 cells.**

| Name | Sequences |
| --- | --- |
| m*Gapdh*_264 (+) | CGGTGCTGAGTATGTCG |
| m*Gapdh*_427 (-) | TGAGTGAGTTGTCATATTTCTCG |
| m*Map7*_983 (+) | TCAAAGCGAGGTCACCG |
| m*Map7*_1136 (-) | CGGATGTTGCCAGGAGA |
| m*Map7d1*_1304 (+) | GCGAACGGAACCTCAAGA |
| m*Map7d1*_1476 (-) | TGGAGGAAGGGCATGTC |
| m*Map7d2*_1098 (+) | TGCAAACGAAGAAACACCAA |
| m*Map7d2*_1257 (-) | CTTATCTGCTACATACTTCTCGG |
| m*Map7d3*_1074 (+) | ATCATTCTCCTTTGGGAGTGTA |
| m*Map7d3*_1252 (-) | CACTTGCTTCAAGGCGT |

**References**

Kikuchi K, Nakamura A, Arata M, Shi D, Nakagawa M, Tanaka T, Uemura T, Fujimori T, Kikuchi A, Uezu A, et al. 2018. Map7/7d1 and dvl form a feedback loop that facilitates microtubule remodeling and wnt5a signaling. EMBO Rep. 19(7):e45471.
